# Case Study of a Comprehensive Team-Based Approach to Increase Colorectal Cancer Screening

**DOI:** 10.13023/jah.0303.07

**Published:** 2021-07-25

**Authors:** Lauren E. Wright, Adam Baus, Andrea Calkins, Holly Hartman-Adams, Mary E. Conn, Susan Eason, Stephenie Kennedy-Rea

**Affiliations:** West Virginia University School of Public Health; WVU Medicine Cheat Lake Physicians; West Virginia University Cancer Institute

**Keywords:** Appalachia, colorectal cancer, cancer prevention, implementation, team-based care, primary care

## Abstract

**Introduction:**

Colorectal cancer is the second leading cause of cancer deaths among men and women in West Virginia. In addition, 51% of all colorectal cancers diagnosed in West Virginia from 2012 to 2016 were detected at either regional (31%) or distant (20%) stages indicating a need for improved early detection.

**Methods:**

West Virginia University Cheat Lake Physicians participated in the West Virginia Program to Increase Colorectal Cancer Screening, a program of Cancer Prevention and Control at the WVU Cancer Institute. As a result, Cheat Lake Physicians assembled a team of health care professionals to implement evidence-based interventions and system changes including provider assessment and feedback, patient reminders, accurate data capture, and tracking of CRC screening tests.

**Results:**

These efforts resulted in a 15.8% increase in colorectal cancer screening rates within one year of implementation. Additionally, the clinic achieved a 66% return rate for Fecal Immunochemical Test kits, an inexpensive, stool-based colorectal cancer screening test.

**Implications:**

The utilization of a team-based approach to patient care yields positive results that can be carried over to other cancer and disease prevention efforts in primary care clinics.

## INTRODUCTION

Colorectal cancer (CRC) is the second leading cause of cancer death among men and women in West Virginia (WV).^1^ In addition, 51% of all colorectal cancers diagnosed in WV were detected at either regional (31%) or distant (20%) stages between the years of 2012–2016.^1^ These statistics underscore the need for early detection through screening. As of 2018, 67% of WV’s eligible population was up-to-date on CRC screening by all U.S. Preventive Services Task Force (USPSTF)–approved screening methods leaving roughly one third of the eligible population unscreened.^2^ CRC incidence rates tend to be higher, and CRC screening rates tend to be lower, in rural areas as compared to urban areas despite the availability of multiple screening modalities.^3^,^4^ Addressing patient barriers improves CRC screening rates, which in turn positively impacts CRC incidence.^5^ A move toward patient-centered care, defined as relationship-based care focused on the whole person, helps clinicians address patient barriers and improve patient outcomes.^6^ Achieving successful patient-centered care requires the creation of an integrated team including physicians, nurses, and clerks who practice team-based care, defined as the provision of healthcare services by at least two collaborating medical professionals for the purpose of assisting their patient in reaching the patient’s health goals.^6^,^7^

West Virginia University (WVU) Cheat Lake Physicians is a primary care clinic located in Morgantown WV that treated approximately 2626 patients aged 50–75 in 2018. Approximately 95% of the clinic’s patients in this age range were insured and were therefore covered 100% for USPSTF-approved screening CRC tests. Cheat Lake Physicians collaborated with the West Virginia Program to Increase Colorectal Cancer Screening (WV PICCS), a Centers for Disease Control and Prevention–funded project administered by Cancer Prevention and Control at the WVU Cancer Institute, which facilitates practice-based change in primary care health systems. A total of 10 primary care clinics in the state of WV were recruited to participate in WV PICCS in 2018 and were collectively referred to as Cohort Year- 3 clinics as they served as the third clinic cohort for the WV PICCS 5-year grant.

The goal of WV PICCS was to increase clinic CRC screening rates by at least 10% above baseline or up to the national goal of 80%. The WV PICCS personnel provided monthly technical assistance with implementation of evidence-based interventions (EBIs), practices that have been shown to positively impact screening rates, starting in January 2018. Cheat Lake Physicians assembled a team, assigned each patient to a primary care provider, created and implemented a client reminder workflow for stool-based CRC screening tests, developed and implemented a CRC screening tracking system, and identified a clinic champion to address the stigma surrounding CRC screening and engage staff and providers. The purpose of this manuscript is to provide an overview of the patient-centered, team-based care Cheat Lake Physicians completed surrounding CRC to serve as a guide for other primary care health systems to follow in their work with quality improvement measures.

## METHODS

### Team Creation and Training

The clinic identified a champion to lead the CRC screening effort and assembled a team consisting of a clinical quality coordinator, a quality improvement coordinator, a provider (champion), a medical assistant, a nurse manager, a lead nurse, and a front desk staff member. This team selected three EBIs to implement in their clinic from a list of CDC-approved EBIs for CRC, developed an implementation plan, and tasked individual team members with the responsibility of relaying implementation plans to respective clinic groups and obtaining their feedback.^8^ The WV PICCS personnel worked with the Cheat Lake Physicians team to provide training sessions to providers and staff. The training sessions focused on CRC, current screening guidelines, communication strategies with patients, and implementation of EBIs. Part of these training sessions included a review of the poor adherence to CRC screening seen in patients who are only offered a colonoscopy and not any of the other USPSTF–approved CRC screening options.^9^ Adherence rate to CRC screening has been found to be more important than screening strategy overall, and Inadomi et al found patients adhered to their CRC screening at a rate of 69% when offered screening options as opposed to 38% when offered colonoscopy only.^9^ Therefore, Cheat Lake Physicians was encouraged to make the offering of USPSTF–approved screening options a priority with their patients.

### Quality Improvement Processes

**Figure 1:**
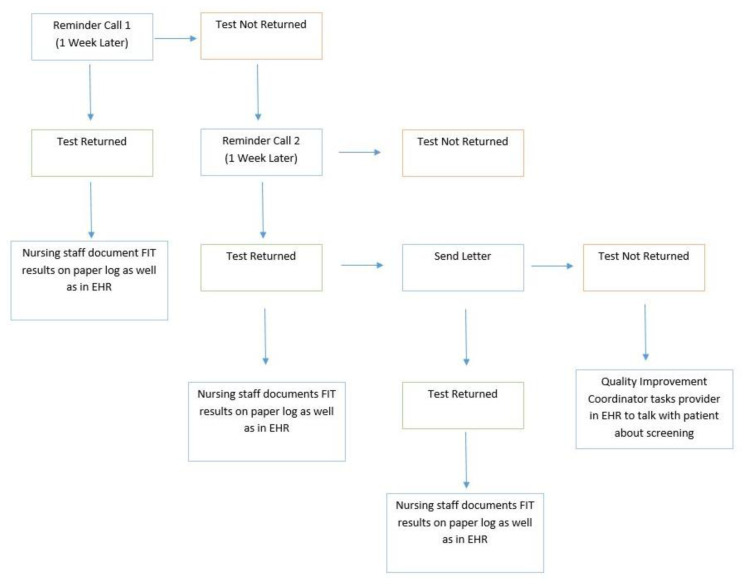
Cheat Lake Physicians Fecal Immunochemical Test (FIT) Call Reminder Program Workflow

Additionally, individual team members contributed ideas regarding new clinic workflows and system changes to implement to meet specified goals. One of the EBIs the team chose was client CRC screening reminders. This involved organizing a workflow for tracking CRC screenings, specifically stool-based screening tests, and calling and sending reminder letters to patients on a regular basis until the patients successfully completed the tests. The quality improvement coordinator developed a reminder workflow that included two phone calls and a letter to patients over the course of a 3-week period (Figure 1).

This workflow focused on FIT kits as they were the most common stool-based screening modality in use at the clinic at the time and because the clinic was responsible for ordering, distributing, and resulting the FIT kits themselves. The workflow for Cologuard™ reminders grew out of the workflow for FIT kits, but this workflow was different due to the fact that Exact Sciences completed reminder calls and letters to patients for 60 days after receipt of the kit. Therefore, the Cologuard™ reminder workflow at Cheat Lake Physicians consisted of reminder letters being sent to patients after the initial 60-day period passed without a screening result obtained. The quality improvement coordinator also created a tracking system in the form of an Excel spreadsheet so that she could follow up with individual patients based on the screening test they received, be that FIT, Cologuard™, or colonoscopy. In conjunction with the call reminder workflow, the quality improvement coordinator created a separate clinic-level tracking system for each screening modality: FIT kits were tracked with order stickers placed in a master binder by nursing staff; Cologuard™ kits were tracked with faxed orders and faxed results both to and from Exact Sciences; and colonoscopies were tracked with printed referrals from the clinic and faxed results from the hospitals. There was no straightforward method by which to complete these activities solely through the electronic health record (EHR), the clinic’s electronic patient record system, which can be viewed as a limitation of the EHR and a potential EHR improvement strategy that is beyond the scope of this manuscript.

A provider served as the clinic champion and collaborated in a variety of activities, both chosen EBIs and other related efforts. The champion participated in monthly team meetings to review implementation plan progress and worked to make changes as needed. The champion also created a sense of healthy competition among the provider/nurse care teams to increase CRC screening rates. The champion fostered a positive work environment for staff and providers by acknowledging individual successes such as when a provider/nurse care team reached a certain milestone in their screening rates or overcame a specific barrier. The champion removed the stigma surrounding CRC screening by creating a comfortable environment for patients. Examples of this included creating t-shirts promoting CRC screening for all the staff, purchasing CRC screening promotional materials such as pens and hats, and utilizing the large inflatable colon model provided by the WV PICCS personnel.

And last, the champion distributed provider assessment and feedback graphs to the other providers during regular provider meetings and used these graphs to foster discussion on successes and challenges through this provision of feedback. The champion also used these graphs to engage providers, to share ideas and challenges, and to keep the providers aware of the importance of focusing on CRC screening.

### Data Assessment and Analysis

A health information technology (HIT) assessment was completed during the clinic’s onboarding process to ensure the data they were pulling and reporting from their EHR, Epic Systems, was accurate. CRC screening is a quality improvement indicator within the EHR meaning that all unscreened patients between the ages of 50–75 have an alert on their patient chart indicating to the provider that a CRC screening is needed. WV PICCS calculated CRC screening rates using a simple percentage calculation with patients in the 50–75 age range with a CRC screening test completed within the recommended time frame included in the numerator and all patients 50–75 included in the denominator. No patients were excluded from the denominator as this was not an option within the EHR. While this is a potential limitation of the EHR, the number of patients who could feasibly be excluded from CRC screening (e.g., terminally ill, no colon) was low enough as to not cause concern among the providers. Baseline CRC screening rate was calculated as the total number of screened patients aged 50–75 years over the total number of patients aged 50–75-years from January 2017 to December 2017. The CRC screening rate at the end of the first implementation year was calculated in the same manner as the baseline rate but for January 2018–December 2018. These rates were then compared to determine if CRC screening rates were increasing as a result of the Cheat Lake Physician team’s efforts.

## RESULTS

**Figure 2:**
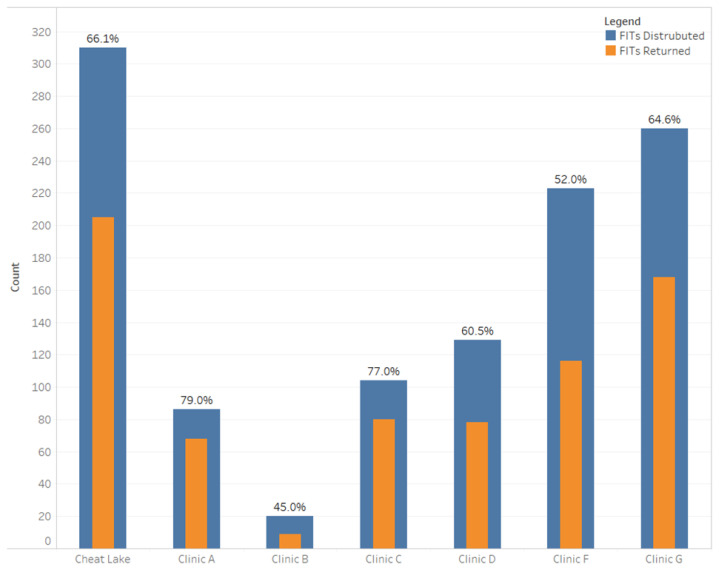
Fecal Immunochemical Test Kit Call Reminder Return Rates for Cheat Lake Physicians as Compared to Other Cohort Year-3 WV PICCS Clinics Participating in the Call Reminder Process

The interventions and methods implemented by the Cheat Lake Physicians team resulted in a variety of changes across the clinic. The clinic delegated the majority of CRC screening responsibilities to nursing staff with the champion spearheading the clinic’s efforts. All nurses were responsible for addressing CRC screening with patients, reviewing both FIT and Cologuard™ kits with patients, and working with their individual providers to decide how best to increase screening rates. This provider/nurse collaboration was created in response to a renewed focus on team-based care and on patient-centered care as the teams functioned for the purpose of providing better care to their patients. Certain nurses were responsible for tracking CRC screening tests, completing patient reminders, organizing clinic educational training sessions, and promoting CRC awareness month. Cheat Lake Physicians empowered their nursing staff to handle CRC screening and other quality improvement measures with a certain amount of autonomy as the nurses had the ability to work well with patients and providers. This increase in autonomy and collaboration among staff resulted in the creation of the FIT call reminder program workflow which contributed to a 66% return rate achieved by the end of the implementation year, meaning that 66% of the FIT kits distributed were returned, representing a comparatively high return rate when compared to other Cohort Year-3 clinics engaged in WV PICCS and contributing to the increase seen in overall CRC screening rates at the clinic (Figure 2). In all Cohort Year-3 clinics, FIT kits were tracked in terms of the number distributed and the number returned within a year. A limitation of this initiative is the lack of baseline FIT kit return rates due to a lack of tracking completed by the clinic prior to the implementation of WV PICCS.

**Figure 3:**
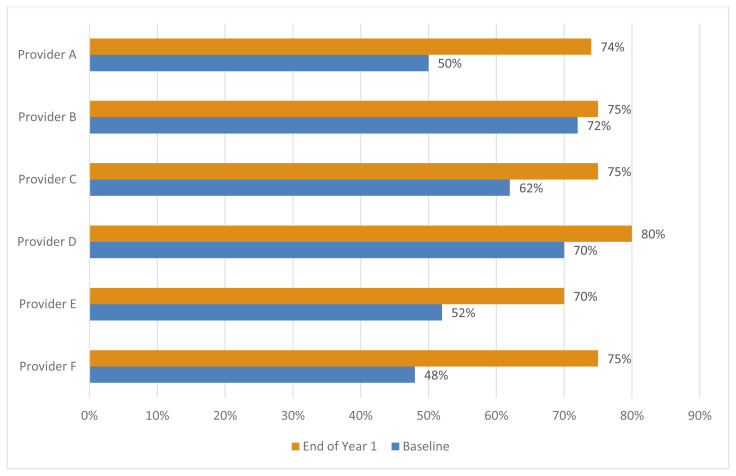
Cheat Lake Physicians Provider-Level Colorectal Cancer Screening Rate Increases from Baseline 2017 to End of Year 2018

Additionally, the team-based approach utilized front desk staff in the process of ensuring patients were assigned to a primary care provider and active in the EHR meaning that a provider was responsible for the quality improvement measures of every patient at the clinic, another result of a focus on patient-centered care. This in addition to the efforts of a dedicated champion resulted in engaged providers who regularly reviewed their performance related to CRC screening and were held accountable by the champion. These provider-related efforts contributed to the overall increase in provider screening rates from 59% at baseline to 74.8% at the end of the implementation year, showing an overall increase of 15.8% (Figure 3).

Cheat Lake Physicians successfully increased CRC screening rates due in large part to their implementation of and commitment to team-based care. Additionally, having an involved and dedicated clinic champion is vital to a successful screening program such as was created at Cheat Lake Physicians.

## IMPLICATIONS

There is currently a focus on redesigning primary care, placing an emphasis on preventive services, team-based approaches, and improving patient-centered care and outcomes.^10^ However, there is a lack of literature available to help guide clinics in implementing a team-based care model leaving clinics without the tools they need to be able to achieve this shift in healthcare design.^11^ Additionally, there is a perception among some providers that team-based care can cause a move away from patient-centered care because of team-based care’s potential to separate care among multiple providers, and this perception has the potential to cause issues in primary care clinics who are encouraged to implement a team-based care model.^6^ Patients are more likely to receive higher quality care if they are being taken care of by a team as opposed to having all responsibility fall to one individual. Recognizing the benefit that nursing and administrative staff can bring to a primary care team could potentially allow for better quality care for patients. Cheat Lake Physicians effectively implemented a patient-centered, team-based approach to primary care that serves as an effective, best practice model for team-based care. Additionally, this success has implications for other cancer and disease prevention efforts in the primary care setting.

**Summary Box**

**What is already known on this topic?** CRC is the second leading cause of cancer-related death in both men and woman combined in WV. Over 51% of WV CRC cases were diagnosed at regional or distant stages in 2012–2016 suggesting the need for on-time CRC screening.

**What is added by this report?** Implementation of a team-based care approach in the primary care setting can significantly increase CRC screening rates.

**What are the implications for future research?** This best practice model for team-based care has implication for other cancer and disease prevention efforts in the primary care setting.
